# Catalytic Hydrolysis of Tricresyl Phosphate by Ruthenium (III) Hydroxide and Iron (III) Hydroxide towards Sensing Application

**DOI:** 10.3390/s20082317

**Published:** 2020-04-18

**Authors:** Lang Zhou, Bryan Chin, Alex L. Simonian

**Affiliations:** Materials Research and Education Center, Department of Mechanical Engineering, Auburn University, Auburn, AL 36849, USA; lzz0028@auburn.edu (L.Z.); chinbry@auburn.edu (B.C.)

**Keywords:** neurotoxin, catalysts, ruthenium (III) hydroxide, iron (III) hydroxide, tricresyl phosphate, gas chromatography–mass spectrometer

## Abstract

Tricresyl phosphate (TCP) is an organophosphorous neurotoxin that has been detected in water, soil and air. Exposure to TCP in cockpit and cabin air poses a severe threat to flight safety and the health of the aircraft cabin occupants. Conventional methods for the detection of TCP in various samples are gas or liquid chromatography coupled to mass spectrometry, which are complex and expensive. To develop a simple low-cost methodology for the real-time monitoring of TCP in the environment, an effective catalyst is demanded for the hydrolysis of TCP under neutral condition. In this study, Ruthenium (III) hydroxide and Iron (III) hydroxide are found to facilitate the production of the alcoholysis and hydrolysis products of TCP, suggesting their role as a catalyst. With this finding, these metal hydroxides provide new potential to realize not only simple colorimetric or electrochemical detection of TCP, but also a simple detoxication strategy for TCP in environment. In addition, the catalytic capability of Ru (III) or Fe (III) hydroxide for TCP gives a hint that they can potentially serve as catalysts for the hydrolysis of alcolyolysis of many other organophosphate compounds.

## 1. Introduction

According to the World Health Report (2003) of the World Health Organization, each year 355,000 people worldwide are killed by unintentional poisonings [1]. The majority of these deaths are strongly associated with excessive exposure to toxic chemicals. Particularly, unawareness of the type of exposure chemicals aggravates the health risks for these chemicals, and delays the damage control and treatment of diseases. Tricresyl phosphate (TCP) is one kind of organophosphate, widely used in industrial applications such as plasticizers, flame retardants and fluid additives. The commercial TCP products consist of various isomers, in which tri-ortho-cresyl phosphate (ToCP) has been proved most neuroxic due to its irreversible damage to a human’s peripheral nerve and spinal cord [2,3,4,5]. ToCP inhibits several vital enzymes, such as acetylcholine esterase [6,7,8] and carboxyesterase [9,10,11]. Acute exposure to ToCP induces nausea, vomiting, diarrhea and abdominal pain, followed by a long asymptomatic period of up to a month. Bilateral degeneration of sensory and motor functions follows acute toxic exposure, and the chance of recovering full motor coordination is poor. Long-term exposure to ToCP can induce a delayed neurodegenerative condition recognized as organophosphorous-induced delayed neuropathy (OPIDN) [12,13,14]. The other isomers of TCP have also been found functional on the inhibition of enzymes in human beings [6,15,16,17,18] and on the formation of axon-like processes, as well as on the disruption of neurofilaments in cultured cells [19].

TCP has been detected widely-distributed in water, soil and air due to its release of production and use [20,21,22]. The presence of TCP and ToCP was reported in cockpits and cabin air [23,24,25], which was likely to cause the symptom clusters of the crew [26]. The conventional methods for detection of TCP in various samples are gas or liquid chromatography coupled to mass spectrometry [27,28,29,30]. These methods are expensive and complicated, and require bulky instruments operated by highly-trained persons, thus there is need for the development of methodology with lowcost portable devices for the real-time monitoring of TCP in samples. To achieve this goal, it is significant to realize the decomposition of TCP first, since TCP itself is difficult to be oxidized or reduced. In previous studies [31,32,33,34], low-cost portable TCP sensors were developed based on the combination of an automatic flow injection system and electrochemical detection, where TCP was rapidly hydrolyzed in pH > 13 alkaline environment (half-life of 1.66 h at pH 13 [35]). Even though the alkaline hydrolysis is here adopted as the simplest way for TCP decomposition, it requires additional steps to introduce alkaline solution, causes alkaline corrosion of device parts in contact, changes the sample pH severely, and produces corrosive wastes. In addition, for the development of other potential TCP sensors, such as colorimetric sensor, the highly alkaline environment required by alkaline hydrolysis is going to inhibit the majority of chromogenic reactions, increasing task difficulty. Meanwhile, the decomposition of TCP plays a vital role in the elimination of TCP in the environment [36]. Its decomposition by alkaline hydrolysis will bring severe pH change, which is harmful to the environment. Therefore, discovering an effective alternative catalyst for TCP hydrolysis or alcoholysis has significance in both the detection and elimination of TCP in the environment. To the best of our knowledge, no catalyst has been reported for the hydrolysis of TCP at a neutral condition.

Fe_2_O_3_ has been reported able to expedite the degradation of several chloroalkyl and alkyl organophosphate flame retardants at circumneutral pH (from half-life > 2 years to < 10 days) [37]. As a Lewis acid, Fe atoms on Fe(OH)_3_ interact with the double-bonded oxygen atom in the phosphate center as a Lewis base, which increases the electrophilicity of phosphate center, making it prone to attack from the H_2_O molecule. Meanwhile, the hydroxyl groups on Fe(OH)_3_ surface play a role in the coordination to the ester functional group, reducing the interaction between the ester bond, making it prone to release [37]. In the Periodic Table of elements, Ruthenium and Iron belong to the same group, Group VIII, thereby Ru(OH)_3_ is expected to share similarity with Fe(OH)_3._ Inspired by this investigation, we explored the efficacy of Fe(OH)_3_ and Ru(OH)_3_ on the degradation of aryl phosphate-TCP. These metal hydroxides with catalytic capability overcome the problems present in alkaline hydrolysis, and provide potential to realize not only simple colorimetric or electrochemical detection of TCP, but also simple detoxication strategy for TCP in the environment.

## 2. Materials and Methods

### 2.1. Reagents and Solutions

P-cresol (Acros Organics, NJ, 99+%) was dissolved in methanol. For the detection of tri-p-cresol phosphate (TCP, Pfaltrz & Bauer, CT) in solution, it was dissolved in methanol. Folin–Denis Reagent was purchased from Sigma-Aldrich (St. Louis, MO, USA). All D.I. water used was from a Millipore Direct-Q water purification system (EMD Millipore, Bedford, MA, USA, with a resistivity of 18 MΩcm^−2^).

### 2.2. Folin–Denis Reaction

Folin–Denis reagent contains the mixture of sodium tungstate and (phospho) molybdic acid in phosphoric acid. To perform the detection of generated cresols in samples, 100 uL TCP sample after metal hydroxide catalysis (step shown below) was mixed with 2 mL 20% Na_2_CO_3_ solution. After incubation of 5 min, 100 uL 0.5X Folin–Denis Reagent was added and reacted for 30 min. The formed colored substance has a maximum absorbance at 720 nm. The UV-VIS spectroscopy (Ultrospec 2100 pro, Amersham Pharmacia Biotech Co., Piscataway, NJ, USA) was obtained at 400 nm~800 nm.

### 2.3. Preparation of Metal Hydroxides

In order to prepare the metal hydroxides, 0.25 mmol CuCl_2_, CoCl_2_, NiCl_2_, MnCl_2_, RuCl_3_, and FeCl_3_ was dissolved in 0.25 mL H_2_O in a EP tube, and 0.75 mL 1M NaOH was added into the dissolved metal ion solution. As precipitates formed immediately, the tube was centrifuged at 12,000 rpm for 10 min. The supernatant was removed and 1 mL 75% methanol/25% H_2_O was added as washing reagent. After uniform dispersion of the precipitates, the same washing procedure as above was repeated three times. At the last time, the supernatant was collected and measured by UV-VIS spectroscopy as control group.

### 2.4. Decomposition of TCP through Alcoholysis

The obtained metal hydroxides from above were dispersed uniformly into 1 mL H_2_O in EP tube, by vigorous vortexing and sonication (10 min). Regarding the suspending liquid, 250 μL was transferred into a new EP tube for centrifugation at 12,000 rpm for 10 min. The supernatant was removed, and the precipitate was added by 1 mL 5 mM TCP dissolved in 75% methanol/25% H_2_O solution. After uniformly mixing by vigorous vortexing, 10 min of reaction time was allowed before the reaction mixture was centrifuged at 12,000 rpm for 10 min. A portion of the supernatant was transferred into a cuvette for direct UV-VIS characterization or Folin–Denis reaction. The remaining supernatant was used for gas chromatography–mass spectrometry (GC-MS, GCT Premier, Waters Corp., Manchester, UK) characterization.

### 2.5. Decomposition of TCP through Hydrolysis

The obtained metal hydroxides from above were dispersed uniformly into 1 mL H_2_O in EP tube, by vigorous vortexing and sonication (10 min). In total, 250 μL of the suspending liquid was transferred into a new EP tube for centrifugation at 12,000 rpm of 10 min. The supernatant was removed, and the precipitate was added with 1 mL 5 mM TCP dissolved in co-solvent of acetone and water. TCP solution was prepared by first dissolving pure TCP into 100% acetone, and then diluted into 5 mM by adding the equivalent H_2_O. After uniformly mixing by vigorous vortexing, 20 min of reaction time was allowed before the reaction mixture was centrifuged at 12,000 rpm for 10 min. The supernatant was withdrawn for gas chromatography–mass spectrometry (GC-MS) characterization.

## 3. Results

### 3.1. UV-Vis Kinetic Characterization of TCP Decomposition

**Figure 1:**
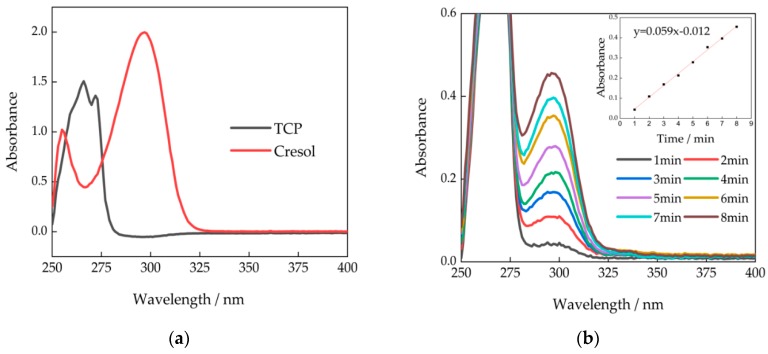
UV-VIS spectrum of alcoholysis profile in (**a**) the standard spectrum of 1mM tricresyl phosphate (TCP) and p-Cresol; (**b**) the dynamic profile of 0.5 mM TCP basic alcoholysis at pH 13.

To investigate the decomposition of TCP via UV-Vis, the UV-Vis absorption spectra of reactant, TCP, and hydrolysis product, p-cresol, were obtained for comparison at 250 nm~400 nm. In the pH 13 co-solvent of 75% methanol and 25% water, 1 mM p-cresol solution displayed the maximum absorption wavelengths at 255 nm and 298 nm, while 1 mM TCP standard solution displayed the maximum absorption wavelengths at 266 nm and 272 nm (Figure 1a). Therefore, the high absorption at 298 nm of p-cresol compared to the low absorption of TCP in this wavelength could be utilized to monitor the production of p-cresol in the process of TCP decomposition. To demonstrate the feasibility of this idea, we monitored the TCP basic alcoholysis process at pH 13 by recording the spectrum of 0.5 mM TCP dissolved in 75% methanol and 25% pH 13 NaOH solution in one minute segments (Figure 1b). The spectra showed a low background absorption at 298 nm at the beginning of reaction, and subsequent linear increase of absorption at 298 nm, indicating the decomposition of TCPs in p-cresols, with a reaction rate of 48 μM/min. Therefore, the observation of decomposition of TCP is enabled by UV-Vis characterization.

### 3.2. Decomposition of TCP by Fe(OH)3 and Ru(OH)_3_

**Figure 2:**
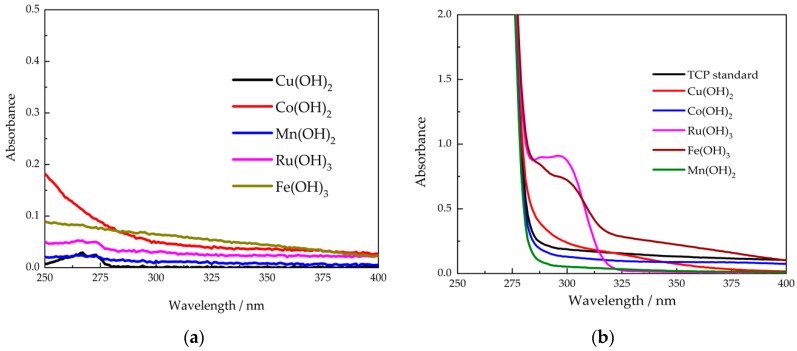
UV-VIS spectrum of (**a**) in the absence and (**b**) in the presence of 5 mM TCP in the case of different metal hydroxides.

Next, we investigated the efficacy of various metal hydroxides on the decomposition of TCP using UV-Vis characterization. First, the supernatants in the last washing step of metal hydroxides were collected for UV-Vis scan. As shown in Figure 2a, no peak was observed at the range of 250 nm~450 nm, suggesting the prepared metal hydroxides did not release any TCP or p-cresols in co-solvent. The metal hydroxides separated from supernatants were then mixed with 5 mM TCP in 75% methanol 25% H_2_O for 10 min of reaction. The absorption spectra of supernatants obtained from centrifugation are shown in Figure 2b. In the absence of metal hydroxides, TCP standard displayed no peak at 298 nm, indicating that no p-cresols were produced, namely, that the decomposition of TCP did not occur. In contrast, a characteristic absorption peak at 298 nm belonging to p-cresol appeared in the presence of Ru(OH)_3_ or Fe(OH)_3_, indicating that TCP had been partially decomposed, and p-cresols had been produced within 10 min. At the same time, in the presence of Cu(OH)_2_, Co(OH)_2_, and Mn(OH)_2_, the supernatants showed similar spectra with TCP standard, and no peak of p-cresols was observed. The results suggested that Fe(OH)_3_ and Ru(OH)_3_ exhibited capability in the decomposition of TCP. Using the calibration curve of standard p-cresol at 298 nm, the quantity of cresols produced in 10 min was calculated as 0.8 mM and 0.5 mM in the presence of Ru(OH)_3_ and Fe(OH)_3_, respectively, which indicated decomposition rates as high as 16% and 10%.

### 3.3. Folin–Denis Reaction of the Decomposition Products

**Figure 3:**
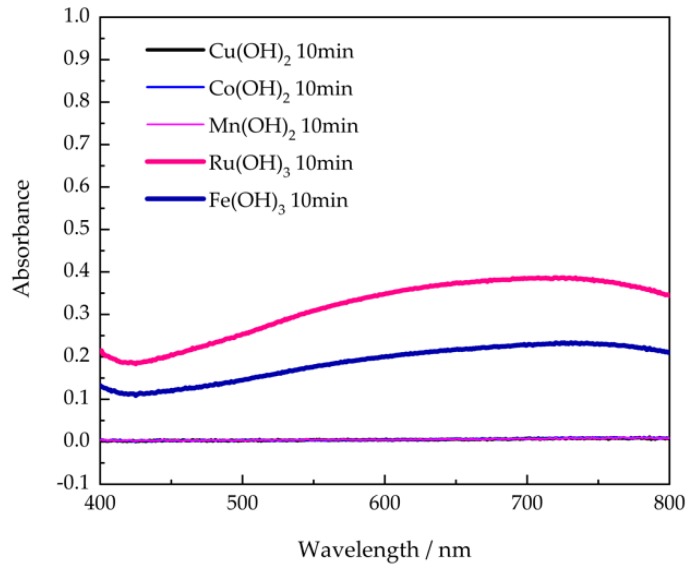
UV-VIS spectrum of resultant solutions after reaction with the Folin–Denis reagent.

To further confirm the production of p-cresols in the presence of Ru(OH)_3_ and Fe(OH)_3_, the Folin–Denis test was performed on the same production solutions seen above in the presence of the metal hydroxides. The UV-Vis absorption spectra in Figure 3 showed that only in the presence of Ru(OH)_3_ and Fe(OH)_3_ the TCP solution display an absorbance peak at 720 nm, which belongs to a blue chromophore constituted by a phenol-phosphotungstic- phosphomolybdenum complex. Compared with the calibration curves of standard p-cresol solutions, the produced cresol had a concentration of 1.3 mM and 0.7 mM for Ru(OH)_3_ and Fe(OH)_3_, respectively. This result validated the production of cresols. The higher value obtained via the Folin–Denis method was presumably due to further decomposition of TCP in basic conditions during the Folin–Denis reaction.

### 3.4. GC-MS Characterization of TCP Alcoholysis

**Figure 4:**
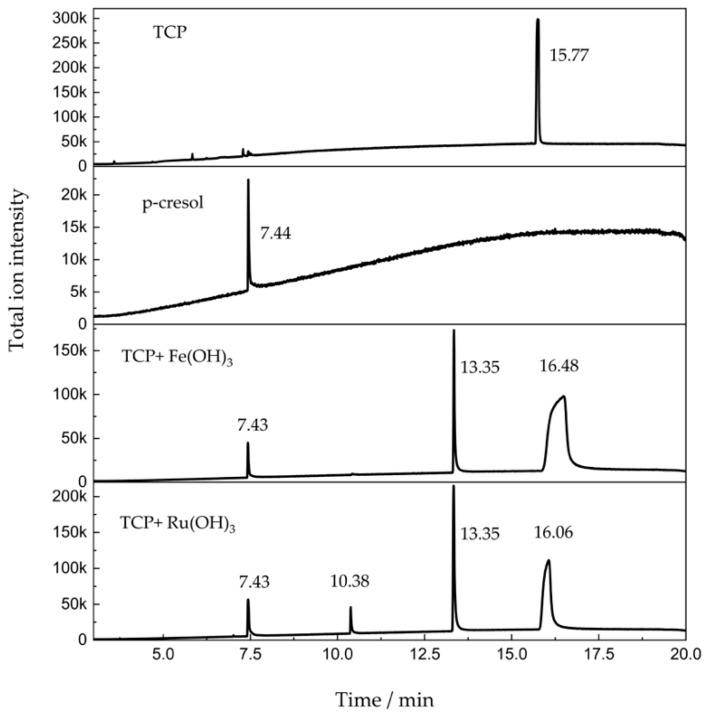
Gas chromatography–mass spectrometer (GC-MS) results for 5 mM TCP after 20 min of reaction with Ru(OH)_3_ and Fe(OH)_3_ in methanol/H_2_O co-Solvent.

The production of p-cresol seen in the above results was likely to result from the alcoholysis or hydrolysis of TCP under the catalysis of Ru(OH)_3_ or Fe(OH)_3_. To confirm this, the solutions after reaction of 20 min from above experiments were characterized by gas chromatography–mass Spectrometer (GC-MS). The product solutions displayed multiple peaks, in which a characteristic product ion m/z= 107 was recognized at a retention time of 7.43 min, m/z= 206.1 was recognized at 10.38 min, m/z= 291 was recognized at 13.3 min, and m/z= 368.1 was recognized at 16 min (Figure 4). Through elemental composition analysis, the peak at 7.43 min belongs to p-cresol, the peak at 10.38 min belongs to cresyl dimethyl phosphate, the peak at 13.3 min belongs to methyl dicresyl phosphate, and the peak at 16 min belongs to tricresyl phosphate (Figure S1). It could be seen that under the catalysis of Ru(OH)_3_, a portion of TCP experienced alcoholysis and released one molecule of cresol, while another portion of TCP released two molecules of cresols. Under the catalysis of Fe(OH)_3_, the hydrolyzed TCP released only one molecule of cresol. Above all, the formation of cresols has been further proven as the result of TCP catalytic alcoholysis.

### 3.5. GC-MS Characterization of TCP Hydrolysis

**Figure 5:**
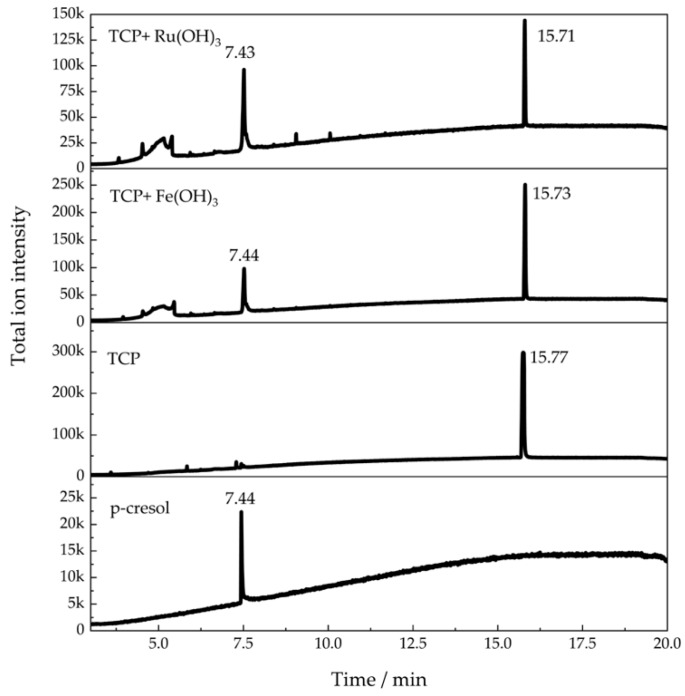
GC-MS results for 5 mM TCP in H_2_O/acetone co-solvent after 20 min reaction in the presence of Ru(OH)_3_ and Fe(OH)_3_.

Followed by the demonstration of catalytic alcoholysis of TCP, its hydrolysis in the presence of Ru(OH)_3_ and Fe(OH)_3_ was also investigated. Acetone was used as an alternate of the cosolvent methanol. Figure 5 shows the formation cresols detected at a retention time of 7.4 min (Figure S2). This indicated that Ru(OH)_3_ and Fe(OH)_3_ were able to catalyze not only the alcoholysis but also the hydrolysis of TCP.

## 4. Conclusions and Discussion

Growing concerns have been raised on the exposure to TCP in the environment, especially in the case of aircraft engine oil leakage-induced inhalation of cabin air. The detection and elimination of TCP in the environment increase the demand for the development of catalysts capable of decomposing TCP at neural pH conditions. In this study, it was found that in the presence of Ru (III) and Fe (III) hydroxide, TCPs were able to decompose through either alcoholysis or hydrolysis into cresols and phosphates, whereas the hydroxides of Cu (II), Co (II), Mn (II) did not display a similar effect. The formation of cresols was validated by UV-VIS spectroscopy, which indicated alcoholysis rates as high as 16% and 10% for Ru (III), and Fe (III) hydroxide, respectively. The formed cresols were further confirmed using Folin–Denis reaction, which showed alcoholysis rates as high as 26% and 14% for Ru (III), and Fe (III) hydroxide, respectively. Analyzed by GC-MS, the products of alcoholysis and hydrolysis of TCP were also validated. Findings from this study suggested a catalytic role of Ru (III), and Fe (III) hydroxide in the decomposition of TCP at circumneutral pH. Ru (III) hydroxide seems to have had a higher catalytic activity compared with Fe (III) hydroxide according to the decomposition rate of TCP.

**Figure 6:**
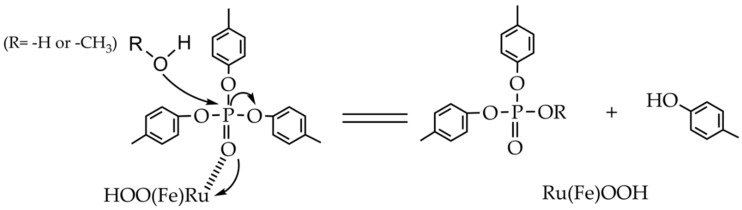
Proposed mechanism of TCP alcoholysis and hydrolysis.

One of the possible mechanisms could be that the oxygen atom as a Lewis base in the phosphate center coordinates with the iron or ruthenium atom as a Lewis acid on the surface of Fe(OH)_3_ or Ru(OH)_3_ (Figure 6). This interaction enhances the attraction of electron clouds away from the phosphorous atom, resulting in its increased electrophilicity. Thus the nucleophilic oxygen atom in water or methanol molecule is allowed to attack the phosphorous atom, leading to the weakened P-O ester bond, and cleavage of the leaving group.

With these findings, new approaches and applications should be expected in both the detection and decontamination of TCP. For instance, a TCP sensor could be potentially realized by integrating Ru (III) or Fe (III) hydroxide and cresol responsive materials such as 2,6-dichloroquinone-4-chloroimide. In the absence of Ru (III) or Fe (III) hydroxide, a rapid hydrolysis of TCP requires a pH above 13, in which 2,6-dichloroquinone-4-chloroimide is not able to react with cresols due to self-decomposition. For another instance, an efficient and simple TCP decontamination system could be built using Ru (III) or Fe (III) hydroxide-packed column, which rapidly hydrolyze TCP in flow and clean the cabin air.

In addition, the efficiency of Ru (III) or Fe (III) hydroxide in catalytic hydrolysis and alcoholysis of TCP provides a hint that they can potentially serve as catalysts for the hydrolysis of alcolyolysis of other organophosphate compounds.

## Supplementary Materials

The following are available online at https://www.mdpi.com/1424-8220/20/8/2317/s1, Figure S1: MS results at various retention time for 1 mM cresols, 5 mM TCP, and its solution after 20 min reaction with Ru(OH)_3_ and Fe(OH)_3_ in alcoholysis 75% methanol/25% H2O; Figure S2: MS results at various retention time for 1mM cresols, 5 mM TCP, and its solution after 20 min reaction with Ru(OH)_3_ and Fe(OH)_3_ in alcoholysis 75% acetone/25% H_2_O.
