# Supplementary material for: Catalytic Hydrolysis of Tricresyl Phosphate by Ruthenium (III) Hydroxide and Iron (III) Hydroxide towards Sensing Application
Source: Sensors (Basel). 2020 Apr 18;20(8):2317. doi: 10.3390/s20082317 (PMC7219232; doi:10.3390/s20082317)
Supplement: Supplementary file 1 [file sensors-20-02317-s001.pdf]

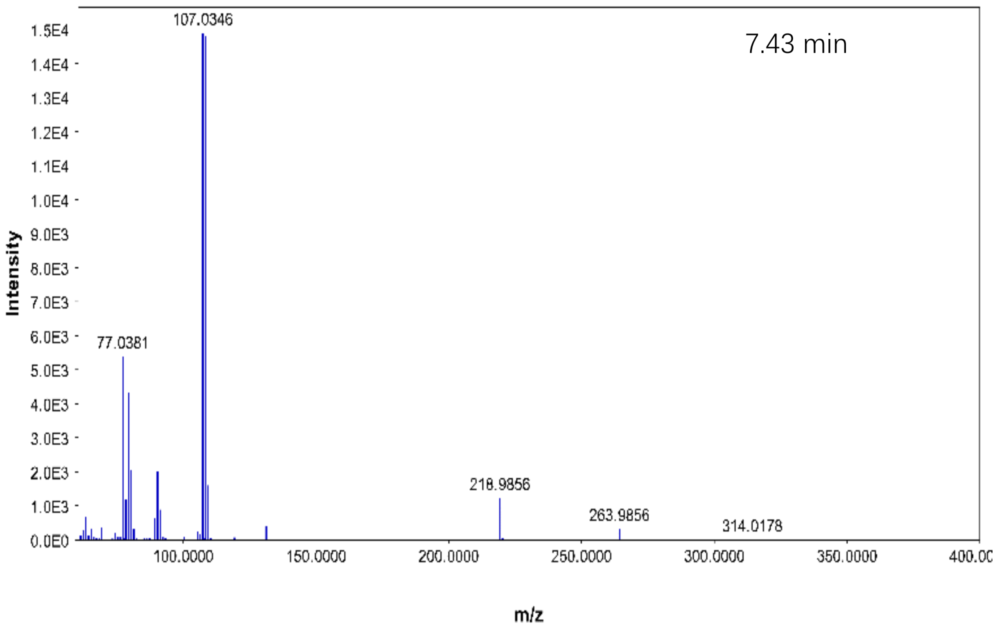

7.43 min
107.0346
77.0381
218.9856
263.9856
314.0178
Intensity
m/z

(**a**)

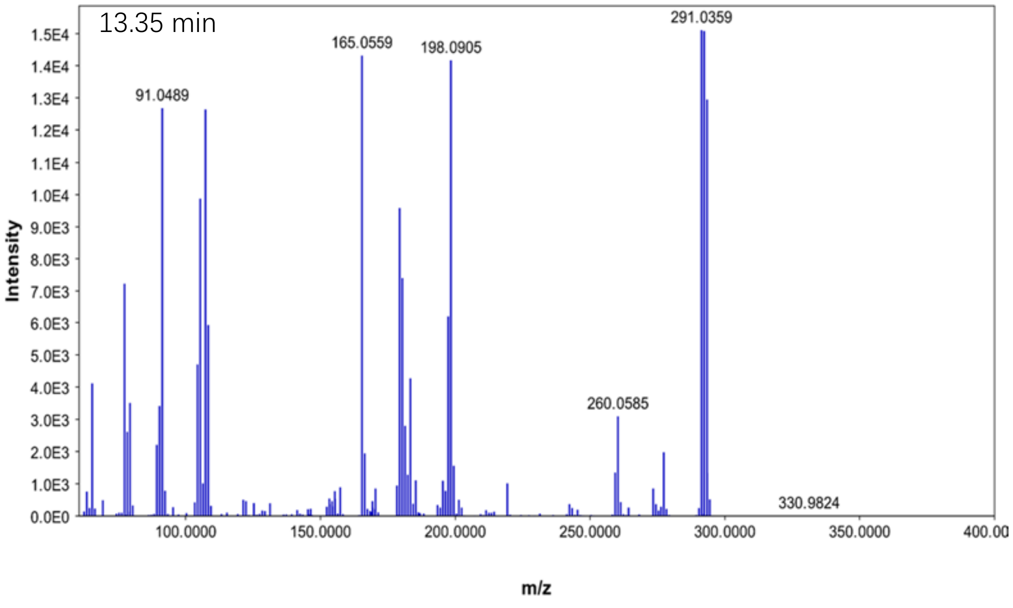

13.35 min
291.0359
165.0559
198.0905
91.0489
260.0585
330.9824
Intensity
m/z

(**b**)

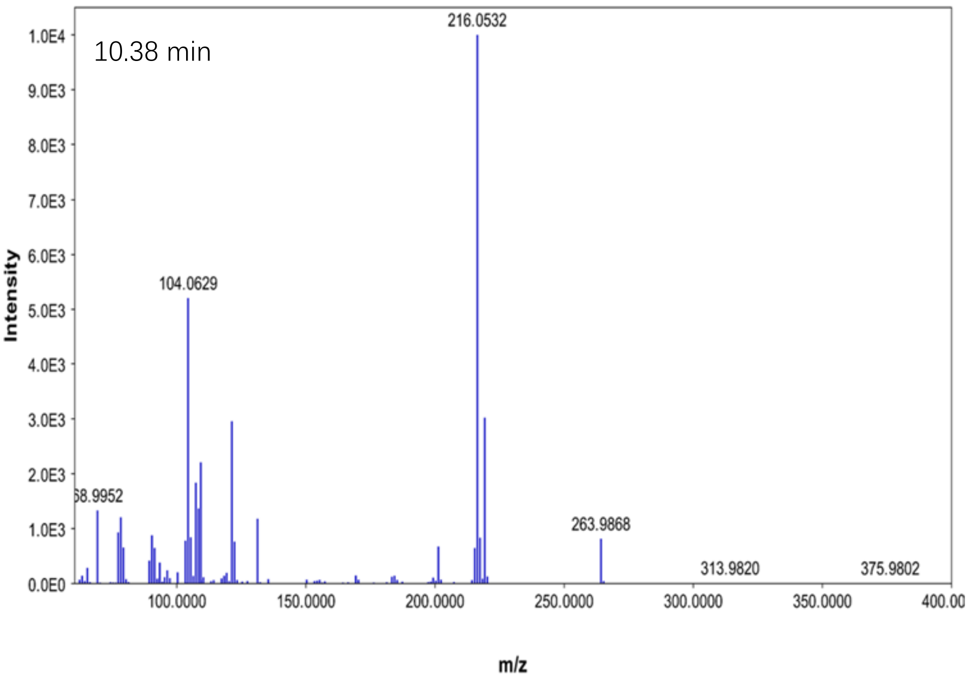

(**c**)

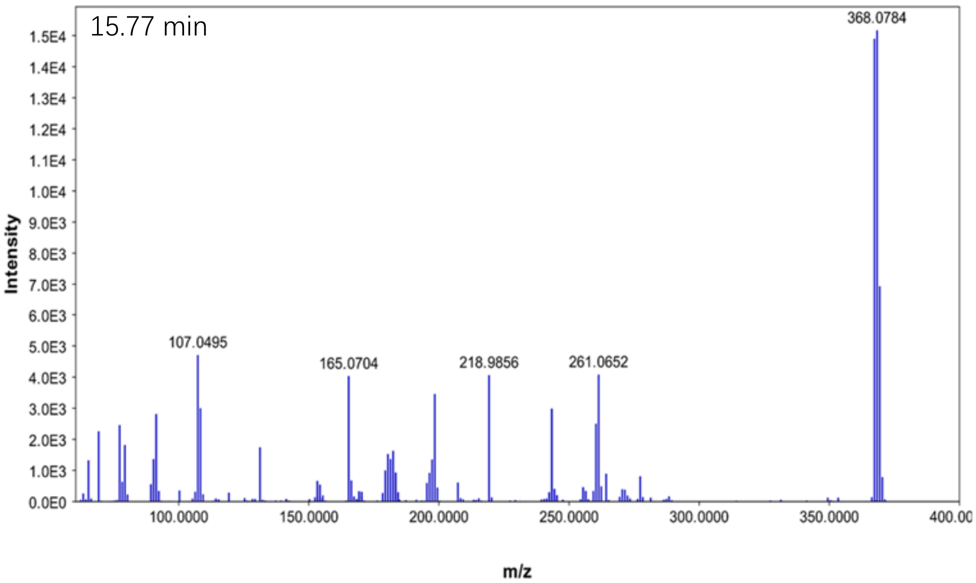

(**d**)

Figure S1. MS results at various retention time for 1mM cresols, 5 mM TCP , and its solution after 20 min reaction with $Ru(OH)_3$ and $Fe(OH)_3$ in alcoholysis 75% methanol/ 25% H_2O.

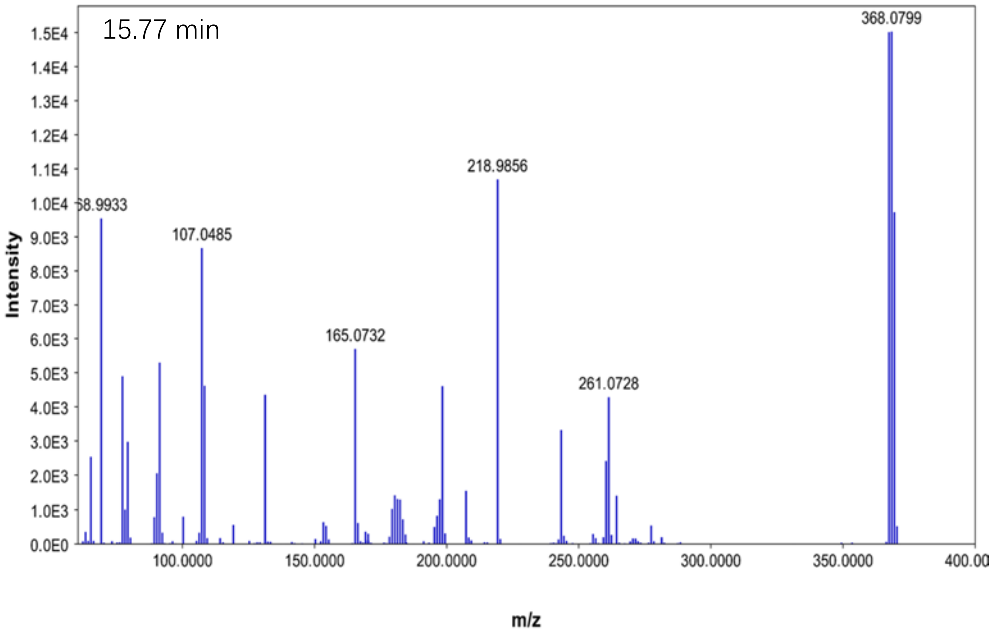

(**a**)

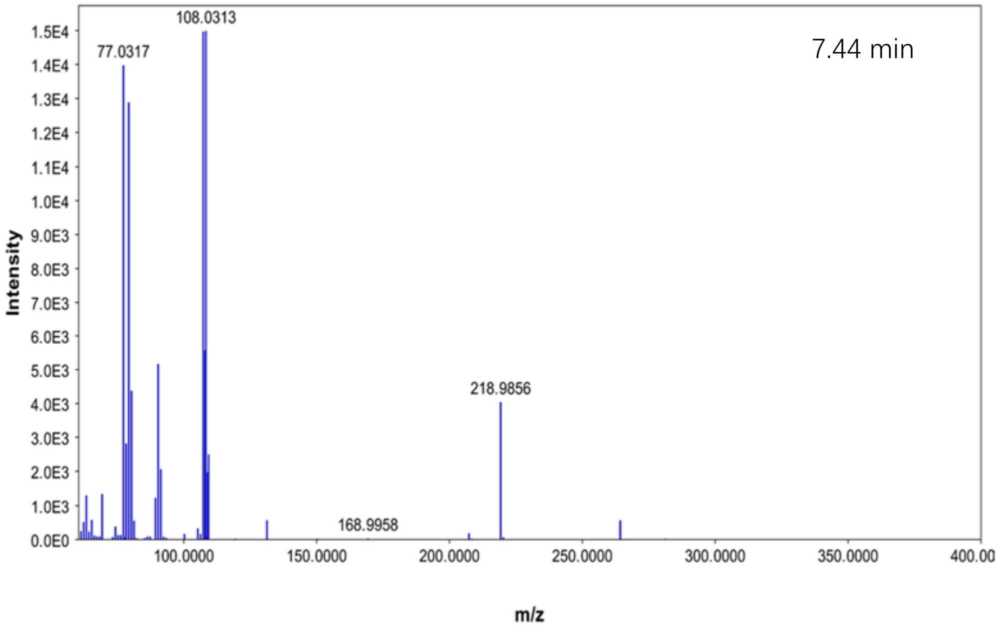

(**b**)

Figure S2. MS results at various retention time for 1mM cresols, 5 mM TCP , and its solution after 20 min reaction with $Ru(OH)_3$ and $Fe(OH)_3$ in alcoholysis 75% acetone/ 25% H_2O.
